# Road to Sustainability: How Can An Academic Journal Contribute to Sustainable Development Goals

**Published:** 2023-03

**Authors:** Iva Grabarić Andonovski

**Affiliations:** Editor of Food Technology and Biotechnology; Secretary of Croatian Association for Scholarly Communication; EASE Council member, Croatian Chapter Chair and Environment and Sustainability Committee Chair; University of Zagreb, Faculty of Food; Technology and Biotechnology,; Pierottijeva 6, 10000 Zagreb, Croatia

The 2030 Agenda for Sustainable Development, adopted at the United Nations Sustainable Development Summit in New York in 2015 by all member states, initiated an action to reach Sustainable Development Goals by 2030, with the aim to protect the planet and its natural resources, end poverty and hunger, build inclusive, just and peaceful societies, reduce inequalities among countries, protect human rights, promote gender equality and reach sustainable economic growth (*1*). The action plan consists of 17 Sustainable Development Goals, namely: 1. no poverty, 2. zero hunger, 3. good health and well-being, 4. quality education, 5. gender equality, 6. clean water and sanitation, 7. affordable and clean energy, 8. decent work and economic growth, 9. industry, innovation and infrastructure, 10. reduced inequalities, 11. sustainable cities and communities, 12. responsible consumption and production, 13. climate action, 14. life below water, 15. life on land, 16. peace, justice and strong institutions, and 17. partnerships for the goals. The progress towards reaching the goals was slow, so in September 2019, the UN Secretary-General called for accelerating sustainable solutions through the Decade of Action plan on three levels: global action, which should provide better leadership, more resources and solutions for SDGs, local action *via* policies, budgets, institutional and governmental support, and people action, creating a momentum by all stakeholders, including academia and societies, pushing for the required transformations (*2*). Covid-19 pandemic set back the efforts made to reach the SDGs, but has shown more than ever the need to dedicate the resources to fight against poverty, inequalities and climate change.

In the run-up to the UN Conference on Sustainable Development Rio+20 in 2012, the Higher Education Sustainability Initiative (HESI) was created, as a partnership between the UN entitites and higher education community (*3*). It has four action groups for sustainable development related to higher education. One of them, SDG Publishers Compact, was launched in 2020 at the Frankfurter Buchmesse by International Publishers Association and UN, with the aim to provide guidelines on how to align education, academic research and publishing with SDGs. So far, 294 publishers (academic institutions, learned societies and publishing houses) and journals have signed the Compact. The signatories commit to the 10 action points, *i.e.* to: 1. show the commitment to the SDGs by stating the sustainability policy and goals and incorporating them into publishing practices, 2. publish and actively promote content that advocates for themes represented by the SDGs, 3. annually report on the progress towards achieving SDGs, 4. nominate a person who is in charge of the SDG progress, 5. raise awareness and promote the SDGs among staff, 6. raise awareness and promote the SDGs among suppliers, 7. advocate SDGs to customers and stakeholders, 8. collaborate with other signatories and organizations, 9. dedicate budget and other resources to achieve SDGs, and 10. take action on at least one SDG (*4*). One of the signatories of the compact, STM, as the leading global trade association for academic and professional publishers, has established the SDG Academic Publishers Forum in 2022, with the aim to provide its members with the tools needed to accelerate the progress in reaching SDGs by 2030 (*5*).

Food Technology and Biotechnology journal signed the UN SDG Publishers Compact on 28 November 2022, to join efforts in reaching SDGs and actively promote environmental awareness and sustainability practices in editing and publishing. The main focus of the journal is on SDG9 (industry, innovation and infrastructure), SDG12 (responsible consumption and production) and SDG13 (climate action), among others. After determining the baseline, the editorial office has undertaken activities promoted in the Environmental sustainability and scientific publishing: EASE manifesto (*6*). Besides signing the Compact, we have appointed a person for monitoring SDG progress and coordinating journal activities, reduced the number of journal printed copies to a required minimum, started to distribute all other material electronically, reduced a number of in-person meetings and travelling to a minimum, switched to energy-efficient appliances and energy-saving LED bulbs, reduced office heating and cooling to a minimum, we recycle more, use only reusable cups and dishes, promote the consumption of locally grown, seasonal food and tap water and usage of public transport, cycling or walking, and enable flexible working hours to reduce commuting during rush hours. Furthermore, we have published the journal environmental policy (*7*). Also, we introduced the option for authors to choose SDG(s) related to their paper, so we can promote them on social networks using SDG nomenclature. In addition, we have established a partnership among the Croatian Association for Scholarly Communication, EASE, HESI and STM, working together on the promotion of SDGs and raising awareness of the importance of sustainable scholarly publishing. Last but not least, we advocate for the change of the journal evaluation criteria developed by our Ministry of Science and Education towards more sustainable and environmentally aware scholarly publishing.

Although they could be seen as small steps in regard to the global crisis, these steps are important in the effort to reach SDGs. Only by joined initiative, scholarly publishing can change to an open and sustainable model that would be of use not only to the scientific, but also to the public community. We would like to see the progress and realization of the 2030 Agenda for Sustainable Development, so we invite you all to join the cause and make a first (however small) step to a new world of scholarly publishing.
